# Advances in AI Technology in Healthcare

**DOI:** 10.3390/bioengineering12050506

**Published:** 2025-05-11

**Authors:** Mohamed Shehata, Mostafa Elhosseini

**Affiliations:** 1 Department of Bioengineering, Speed School of Engineering, University of Louisville, Louisville, KY 40292, USA; 2 Computers and Control Systems Engineering Department, Faculty of Engineering, Mansoura University, Mansoura 35516, Egypt; 3 College of Computer Science and Engineering, Taibah University, Yanbu 46421, Saudi Arabia

## 1. Introduction

This Special Issue unites 11 innovative research papers that study artificial intelligence applications in the fields of bioengineering and healthcare. The research contained herein demonstrates how AI technology advances through its applications, which range from assistive technologies for disabled people to machine learning models that predict and diagnose diseases. The research studies included in this Special Issue demonstrate how AI technology advances healthcare transformation, while also exploring patient safety, medical diagnostics, ethical considerations, and social effects. This Special Issue features essential research demonstrating how AI technology can enhance healthcare results through individualized, efficient, and accessible medical interventions.

## 2. Key Research Contributions

- Assistive technologies for disabilities: Puthu Vedu et al. [1] proposed a new tactile learning tool for visually and hearing-impaired people, which used 3D convolutional neural networks (CNNs) and bidirectional LSTM networks to convert spoken words into Morse code for communication through a wearable device. This tool enhanced the learning experience of deafblind students by offering them a more effective means of communication, which went beyond the traditional tactile methods.
- Health-related quality of life prediction: Abegaz et al. [2] used machine learning algorithms to predict health-related quality of life (HRQOL) based on social determinants of health (SDOH). The study’s findings show that non-medical factors, such as social, educational, and environmental variables, are important in determining overall health outcomes, thus offering a new approach for personalized healthcare that includes a wider range of determinants.
- Surgical instrument recognition: Haider et al. [3] assessed the performance of ChatGPT-4o and other specialized mobile applications for recognizing surgical instruments using large language models (LLMs). It is apparent from their study that while LLMs do effectively classify instruments, they still struggle with subtype distinctions. These findings stress the possibility of using AI-powered technologies to automate surgical instrument control, thus improving patient protection during procedures.
- Coronary artery disease: Wang et al. [4] examined the co-occurrence patterns of comorbidities and diagnoses in patients with coronary artery disease (CAD) through network analysis. They picked up on hypertension as an intermediate node where unstable angina and myocardial infarction often co-occurred with metabolic diseases. There were sex- and age-based differences for which there needs to be individualized treatment, as well as further studies.
- Cervical cancer prediction: ViT-PSO-SVM, a recent method by AlMohimeed et al. [5], integrated vision transformers (ViT) with particle swarm optimization (PSO) and support vector machines (SVM) to detect cervical cancer precisely. This method provides exceptional performance, specifically with respect to cervical cancer classification from image datasets, providing a strong, non-invasive diagnosis tool for early detection.
- Skin disease diagnosis: Malik et al. [6] designed a deep learning-based skin disease diagnosis platform with 87.64% accuracy based on a convolutional neural network (CNN) from dermoscopic images. This platform is a vital breakthrough in dermatology diagnosis when compared to the conventional method, providing a reliable means of offering real-time diagnosis in clinical practice.
- Cost-effective medicine analysis: Machine learning was employed by Long et al. [7] to uncover key drivers for the cost-effectiveness of over-the-counter medicines. According to their study, products that qualify as flexible spending accounts (FSAs) or health savings accounts (HSAs), medicines, products that cure a wider set of symptoms, and products with tiny packaging are seen as more cost-effective. They can assist customers and manufacturers in making better purchasing and marketing decisions based on knowledge when consumers and firms make decisions.
- Ventricular dysfunction detection: Makimoto et al. [8] utilized deep learning models in recognizing ventricular dysfunction from electrocardiograms (ECGs). The test indicates that the artificial intelligence models developed can enhance diagnostics to become more precise, especially when handling two-beat ECGs, thus presenting a superior instrument with which to identify heart diseases and aid clinicians in better decision-making.
- Kidney volume measurement in ADPKD: Hsu et al. [9] applied deep learning models for the precise measurement of total kidney volume (TKV) in patients with autosomal dominant polycystic kidney disease (ADPKD) from MRI scans. The results indicate that medical experts are not as capable as deep learning models, which deliver a reliable, non-invasive method for estimating the level of disease and better patient care.
- Cervical cancer screening using pap smears: Ando et al. [10] proposed an explainable deep learning method for the cervical cancer screening of pap smear images. Their OCC- and VAE-based method discriminates between normal and abnormal cells using unlabeled abnormalities and is thus a strong candidate for use in automated, trustworthy cervical cancer screening.
- AI support for informal caregivers: Borna et al. [11] conducted a systematic review of AI contributions to informal patient caregiving assistance. The findings of their review establish the potential of AI-based applications to minimize caregiver burden, enhance efficiency, and support caregiver health. Regardless of variations in methodology, the studies reviewed consistently indicate that AI can provide adaptive and intelligent support to caregivers.

## 3. Trends and Insights

Among the overlying themes throughout these studies is the expanding utilization of AI-driven models for the automation and enhancement of diagnostic protocols, especially in applications such as healthcare and disease prognosis. A number of these studies—for instance, those conducted by Abegaz et al. [2] and AlMohimeed et al. [5]—refer to the use of multisource datasets, such as those pertaining to the social determinants of health and medical imaging, for the improvement of the accuracy and reliability of predictions. Also, the use of explainable AI (XAI) methods, such as GradCAM employed by ViT-PSO-SVM, is one aspect of an overall trend toward making AI models more interpretable and transparent, which is important for clinical application and adoption.

Moreover, there is also a clear direction towards multimodal AI applications that combine different modes of data (e.g., images, text, and physiological signals) for improving diagnostic accuracy. For instance, Haider et al. [3] and Malik et al. [6] describe how AI models are able to analyze different medical images to detect diseases, while Puthu Vedu et al. [1] illustrate how AI can facilitate the bridging of the communication barrier for people with multiple disabilities via wearable devices.

## 4. Challenges and Gaps

Although encouraging in their results, there are still some pending issues. One impeding factor shared across these studies is the absence of standardized data structures for AI models, hindering AI systems in their smooth adaptation across various healthcare environments. Haider et al. [3] state variability in model performance when used across various instruments or classes, highlighting the need for larger and more uniform training data. Likewise, Hsu et al. [9] illustrate how image orientation in MRI imaging adds biases that affect the overall generalizability of AI models in clinical settings.

Apart from that, although the performance of such AI models is extremely high in controlled environments, the applicability of such tools for use in real-world practice is unclear. Malik et al. [6] quote the finding that the CNN model’s accuracy in identifying skin diseases is acceptable, but clinical validation will be necessary in order to evaluate its use in real-life practices in mixed-patient populations.

## 5. Future Directions

There are only a few avenues for possible future research. First, there is a need for multicenter and longitudinal studies based on various datasets and various patient populations to validate the AI models presented in these papers. For example, Abegaz et al. [2] need to extrapolate their HRQOL predictive models to diverse patient populations to make the evidence more generalizable. In addition, Haider et al. [3] also suggest adding multimodal information—i.e., operating room videos in real time and audio signals—to surgical instrument detection systems to improve their accuracy.

Another important direction is integrating AI clinical workflows. Future research has to be aimed at understanding how AI technologies can be embedded in current healthcare systems such that workflow efficiency and patient outcomes can be maximized. AlMohimeed et al. [5] suggest enlarging their ViT-PSO-SVM model to the remaining image modalities, while Malik et al. [6] propose using their skin disease diagnosis system in real-world clinical settings.

Lastly, ethical concerns will remain an area of focus. As more and more AI tools are being applied, ensuring transparency, fairness, and accountability in such models will be the key to gaining the trust of healthcare professionals and patients alike. This is especially true in caregiving scenarios, wherein the capacity of AI to enhance informal caregiving is conceived by Borna et al. (2024) [11] as an application with immense potential to contribute to improving the standard of care, as well as the quality of life for both patients and caregivers.

## 6. Conclusions

This Special Issue presents an extensive review of the impact of AI in bioengineering and healthcare. These studies not only identify the technological leaps in disease diagnosis, surgical assistance, and assistive technology but also indicate challenges and future research directions with respect to applying these technologies in the clinical environment. With the capacity to improve diagnostic accuracy to support both better patient care and caregiver guidance, AI is poised to revolutionize healthcare delivery. However, sustained research in many inter-related domains, such as data standardization, real-world validation, and ethical artificial intelligence, is needed in order to achieve this potential. Subsequent studies are encouraged to employ these findings to develop more effective, accessible, and resilient AI-based healthcare technologies.

## Data Availability

Not applicable.

## References

[1] Puthu Vedu S.Z., Altulyan M., Singh P.K. (2025). A Novel Tactile Learning Assistive Tool for the Visually and Hearing Impaired with 3D-CNN and Bidirectional LSTM Leveraging Morse Code Technology. Bioengineering.

[2] Abegaz T.M., Ahmed M., Ali A.A., Bhagavathula A.S. (2025). Predicting Health-Related Quality of Life Using Social Determinants of Health: A Machine Learning Approach with the All of Us Cohort. Bioengineering.

[3] Haider S.A., Ho O.A., Borna S., Gomez-Cabello C.A., Pressman S.M., Cole D., Sehgal A., Leibovich B.C., Forte A.J. (2025). Use of Multimodal Artificial Intelligence in Surgical Instrument Recognition. Bioengineering.

[4] Wang J., Qi Z., Liu X., Li X., Cao Z., Zeng D.D., Wang H. (2024). Population and Co-Occurrence Characteristics of Diagnoses and Comorbidities in Coronary Artery Disease Patients: A Case Study from a Hospital in Guangxi, China. Bioengineering.

[5] Makimoto H., Okatani T., Suganuma M., Kabutoya T., Kohro T., Agata Y., Ogata Y., Harada K., Llubani R., Bejinariu A. (2024). Identifying Ventricular Dysfunction Indicators in Electrocardiograms via Artificial Intelligence-Driven Analysis. Bioengineering.

[6] Hsu J.L., Singaravelan A., Lai C.Y., Li Z.L., Lin C.N., Wu W.S., Kao T.W., Chu P.L. (2024). Applying a Deep Learning Model for Total Kidney Volume Measurement in Autosomal Dominant Polycystic Kidney Disease. Bioengineering.

[7] Malik S.G., Jamil S.S., Aziz A., Ullah S., Ullah I., Abohashrh M. (2024). High-Precision Skin Disease Diagnosis Through Deep Learning on Dermoscopic Images. Bioengineering.

[8] Long B., Zhou J., Tan F., Bellur S. (2024). Deciphering Factors Contributing to Cost-Effective Medicine Using Machine Learning. Bioengineering.

[9] AlMohimeed A., Shehata M., El-Rashidy N., Mostafa S., Samy Talaat A., Saleh H. (2024). ViT-PSO-SVM: Cervical Cancer Prediction Based on Integrating Vision Transformer with Particle Swarm Optimization and Support Vector Machine. Bioengineering.

[10] Ando Y., Cho J., Park N.J.Y., Ko S., Han H. (2024). Toward Interpretable Cell Image Representation and Abnormality Scoring for Cervical Cancer Screening Using Pap Smears. Bioengineering.

[11] Borna S., Maniaci M.J., Haider C.R., Gomez-Cabello C.A., Pressman S.M., Haider S.A., Demaerschalk B.M., Cowart J.B., Forte A.J. (2024). Artificial Intelligence Support for Informal Patient Caregivers: A Systematic Review. Bioengineering.

